# Investigating Floating-Gate Topology Influence on van der Waals Memory Performance

**DOI:** 10.3390/nano15090666

**Published:** 2025-04-27

**Authors:** Hao Zheng, Yusang Qin, Caifang Gao, Junyi Fang, Yifeng Zou, Mengjiao Li, Jianhua Zhang

**Affiliations:** School of Microelectronics, Shanghai University, Jiading, Shanghai 201800, China; 2022@shu.edu.cn (H.Z.); 2130920057@shu.edu.cn (J.F.); zouyifeng2001@163.com (Y.Z.);

**Keywords:** van der Waals transistor, floating-gate memory, memory window, electrostatic coupling

## Abstract

As a critical storage technology, the material selection and structural design of flash memory devices are pivotal to their storage density and operational characteristics. Although van der Waals materials can potentially take over the scaling roadmap of silicon-based technologies, the scaling mechanisms and optimization principles at low-dimensional scales remain to be systematically unveiled. In this study, we experimentally demonstrated that the floating-gate length can significantly affect the memory window characteristics of memory devices. Experiments involving various floating-gate and tunneling-layer configurations, combined with TCAD simulations, were conducted to reveal the electrostatic coupling behaviors between floating gate and source/drain electrodes during shaping of the charge storage capabilities. Fundamental performance characteristics of the designed memory devices, including a large memory ratio (82.25%), good retention (>50,000 s, 8 states), and considerable endurance characteristics (>2000 cycles), further validate the role of floating-gate topological structures in manipulating low-dimensional memory devices, offering valuable insights to drive the development of next-generation memory technologies.

## 1. Introduction

The rapid advancement of artificial intelligence and big data has driven the demand for high-density, high-performance storage. This makes the scaling of memory devices inevitable, although they do not directly face the same severe scaling challenges as transistors [1,2]. Floating-gate memory (FGM), as a key commercial storage technology, has achieved significant milestones in certain areas, for example, Micron and Intel have adopted 3D NAND technology with floating-gate cells, offering about three times the storage capacity of conventional NAND dies [3]. However, the ITRS technology roadmap predicts severe challenges for FGM scaling beyond the 12-nm node [4]. Emerging van der Waals (vdWs) materials provide viable solutions for further scaling [5,6,7,8]. The introduction of vdWs into FGM devices not only enables a minimized device design of the channel, but also offers an ideal platform for in-depth studies of the physical properties of memory device due to their back end of line (BEOL) processing compatibility and immunity to the short channel effect [9,10,11,12,13,14,15,16,17,18,19,20,21,22,23]. For example, graphene (Gr) as the floating-gate layer can maintain an extremely high current density with effective protection against gate oxide layer contamination and suppress ballistic current-induced charge storage degradation compared with its polycrystalline silicon counterparts [24,25,26,27,28,29,30,31,32,33,34,35,36]. In particular, with device dimensions stepping into the nanoscale, the floating-gate configuration has become more critical, as it dominates the storage capability such as storage states and retention [32,37,38]. Although the relevant feature between the floating gate and back gate in vdWs FGM has been mentioned, systematic research on the coupling effects among critical terminals remains relatively scarce, with many key issues yet to be thoroughly explored and resolved [28,39]. Consequently, more efforts should focus on in-depth investigations into the effect of floating-gate configurations on storage characteristics to achieve high-performance memory devices.

In this study, we systematically investigated the floating gate-topology-dependent memory behavior of typical vdWs FGM devices by combining experimental and theoretical approaches. The fabricated MoS_2_ FGM with a longer floating gate demonstrated better carrier storage capability and delivered a positive correlation between the memory window (MW) and the floating-gate length. A saturation phenomenon of the MW could be observed by either prolonging the floating-gate layer over the channel length or increasing the thickness of the tunneling layers, which emphasizes the critical role of floating-gate design in manipulating the charge storage characteristic in FGM devices. TCAD device simulation was further conducted to clarify the floating-gate configuration-dependent electrical field distribution for carrier tunneling and the negative effect of electrostatic coupling between the floating gate and source/drain (S/D) electrodes on the carrier tunneling process. Based on the optimized device design, we further performed fundamental device characterization and achieved a good memory performance including a large memory window ratio (82.25%), good retention (>50,000 s, 8 states), and considerable endurance characteristics (>2000 cycles). This work establishes a connection between floating-gate design and memory performance in vdWs FGM devices, providing insights for further scaling and future developments.

## 2. Experimental

This study fabricated a series of vdWs FGM devices using a back-gate structure on a SiO_2_/Si substrate with a 300 nm SiO_2_ layer as the gate dielectric.

2H-MoS_2_ flakes were employed as the semiconductor channel. Few-layer Gr and h-BN with different sizes or thicknesses were used to serve as the floating gate and tunneling layer, respectively.

The fabrication process begins with the thermal evaporation of a pair of 50 nm thick Au electrodes onto a cleaned SiO_2_/Si substrate. Gr, h-BN, and MoS_2_ flakes are then mechanically exfoliated using PDMS films and sequentially transferred onto the target substrate [10]. The Gr floating gate is entirely encapsulated with h-BN layers, which serve as a tunnel barrier, isolating the channel from the MoS_2_ channel. The Gr floating gate is laterally encapsulated by h-BN and remains electrically isolated from both the MoS_2_ channel and control gate. This design ensures charge confinement while enabling field-driven charge injection. Finally, a few-layer Gr is aligned and transferred to the ends of the MoS_2_ films to serve as source and drain electrodes with a fixed channel length of 15 μm, completing the device fabrication. During this process, vdWs materials are screened using an optical microscope to ensure that their thickness meets the experimental requirements [9]. Electrical measurements were conducted in a nitrogen atmosphere at 300 K using a Keysight B2912B semiconductor parameter analyzer, (Krysight, Bayan Lepas, Malysia) effectively minimizing environmental interference and ensuring stable device characterization. To further investigate the device structure and surface morphology, atomic force microscopy analysis (AFM) was performed to examine the thickness of fabricated devices across different materials [5]. Additionally, device simulations were performed using Silvaco TCAD 2021, providing a theoretical framework to analyze device behavior.

## 3. Results and Discussion

**Figure 1:**
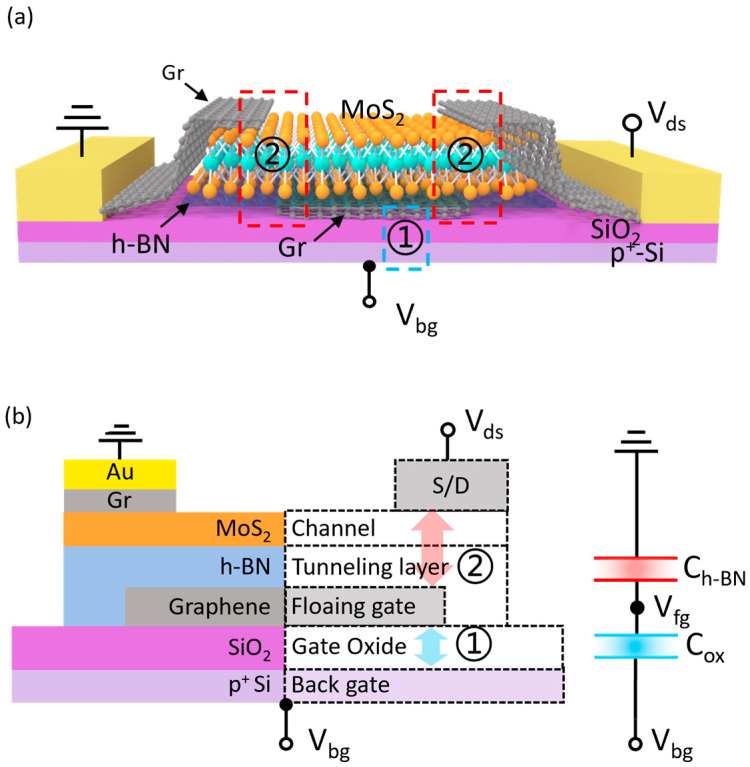
(**a**) Three-dimensional structural schematic of a typical vdWs FGM, where the blue box represents the coupling effect between the floating gate and the back gate, and the red box represents the coupling behavior between the floating gate and the S/D electrodes. (**b**) Two-dimensional structural schematic of the vdWs FGM with ① Denotes the coupling between the back gate and the floating gate. ② Denotes the coupling between the floating gate and the source/drain terminals interrelations among critical terminals and the equivalent circuit with the main capacitances.

In general, the equivalent circuit of a typical vdWs FGM can be represented as a series connection of Cox and Ch-BN, as illustrated in Figure 1, where Cox denotes the capacitance between the back gate and the floating gate, and Ch-BN represents the capacitance between the floating gate and the S/D electrodes. It is evident that the topology of the floating-gate layer plays a crucial role in shaping the performance of the vdWs FGM. The coupling behavior between the back gate and the floating gate has been basically evaluated in existing studies [40], however, the interrelations between the floating gate and other critical terminals, such as the source and drain electrodes, remain unclear.

**Figure 2:**
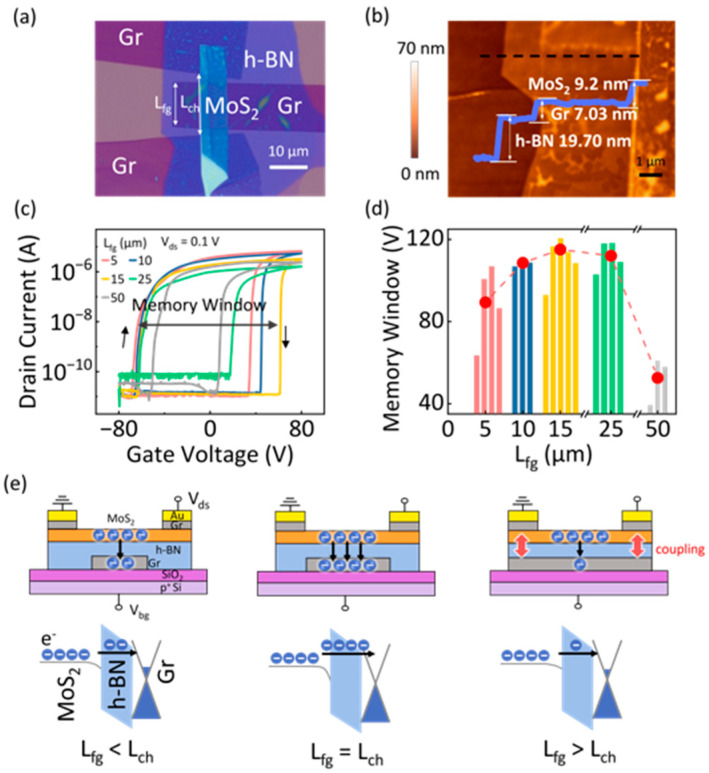
(**a**) A typical OM image of the vdWs FGM. (**b**) AFM image and the corresponding height profile of the fabricated device. (**c**) Transfer curves for devices with an L_fg_ of 5, 10, 15, 25, and 50 μm, measured under a fixed V_ds_ of 0.1 V. (**d**) Histogram of MW for devices with varying L_fg_. The dashed line with red dots shows the average values of MW. (**e**) Schematic of the working mechanisms and electron transport behavior with different L_fg_.

Therefore, to systematically investigate the influence of floating-gate topology on memory performance, devices with an elaborate design, for example, by varying the floating-gate length and the tunneling-layer thickness, were experimentally fabricated. At first, by adjusting the floating-gate length (L_fg_), this study explored how structural variations impact the charge storage and MW characteristics. In this device series, the channel length was maintained at 15 µm, while the L_fg_ was varied symmetrically with respect to the S/D electrodes. The optical microscope (OM) image of a representative device is shown in Figure 2a, providing a clear view of the material stacking. Additionally, AFM was used to determine the thicknesses of the key layers in the device structure. As shown in Figure 2b, the thicknesses of MoS_2_, h-BN, and Gr flakes were 9.20 nm, 19.70 nm, and 7.03 nm, respectively. The output characteristic curves of the FGM device exhibited excellent linearity (Figure S1), indicating that the Gr S/D electrodes formed a high-quality ohmic contact with the MoS_2_ channel. Furthermore, Raman spectroscopy analysis demonstrated the high-quality vertical stacking of the constituent material layers in the FGM heterostructure (Figure S2). To evaluate the impact of L_fg_ on memory behavior, electrical measurements were performed under a ±80 V bidirectional back gate voltage (V_bg_) sweep with a fixed drain voltage (V_ds_) of 0.1 V. As shown in Figure 2c, the measured transfer curves revealed significant variations in MW as the L_fg_ changed. The varying trend could be further visualized in the statistical results of 19 devices (Figure 2d). Initially, as the L_fg_ increased, the MW expanded, indicating enhanced charge storage capacity. However, when the L_fg_ approached or exceeded the channel length (L_ch_), a saturation phenomenon occurred, followed by a gradual MW decline. The working mechanism of the FGM devices was hypothesized to explain this phenomenon, as illustrated in Figure 2e. For devices with shorter floating gates, increasing the L_fg_ enhances the charge storage capability, leading to a gradual MW increase. However, as the L_fg_ extends closer to the S/D electrodes, electrostatic coupling intensifies, weakening the vertical electric field strength between the floating gate and channel, thereby hindering the electron tunneling process. Consequently, MW exhibits a saturation trend before gradually decreasing. These findings emphasize the crucial role of floating-gate topology in shaping memory performance in low-dimensional FGM devices [40].

**Figure 3:**
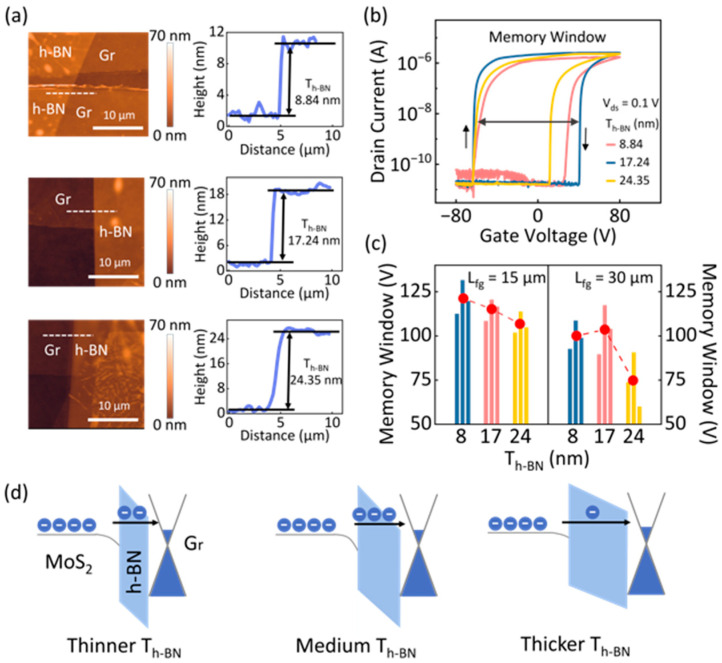
(**a**) A typical AFM image of the vdWs FGM with different T_h-BN_ with L_fg_ = 30 μm. (**b**) Transfer curves for devices with varying T_h-BN_ at L_fg_ = 30 μm, measured under a fixed V_ds_ of 0.1 V. (**c**) Histogram of MW for devices with varying T_h-BN_ at L_fg_ = 15 and L_fg_ = 30 μm, respectively. The dashed lines with red dots represent the average values of MW. (**d**) Schematic of the working mechanisms and electron transport behavior with different T_h-BN_.

To further verify the above-mentioned hypothesis regarding the influence of electrostatic coupling between the floating gate and S/D electrodes, an in-depth investigation was performed on the charge storage characteristics by tuning the carrier tunneling behavior. Specifically, by systematically varying the thickness of the h-BN tunneling layer (T_h-BN_), how the floating-gate configuration impacts MW, particularly in the presence of electrostatic coupling effects, can be determined. For a fair comparison, two sets of devices were fabricated with the L_fg_ fixed at 15 μm and 30 μm. For each set, three different T_h-BN_ were adopted, which were categorized as the thinner, medium, and thicker h-BN layer. The specific three thicknesses, such as 8.84 nm, 17.24 nm, and 24.35 nm, were further clarified using AFM analysis (Figure 3a). As a result, the measured transfer curves in Figure 3b clearly illustrated significant variations in MW as the T_h-BN_ changed. The extracted MW trends are summarized in Figure 3c, revealing distinct behaviors for the two L_fg_ sets. Specifically, for devices with L_fg_ = 15 μm, MW exhibited a monotonic decrease as the T_h-BN_ increased. In contrast, for devices with L_fg_ = 30 μm, MW followed a hump-shaped trend, initially increasing before gradually declining as the T_h-BN_ continued to rise. The physical mechanisms underlying this behavior are illustrated in Figure 3d. Taking devices with a longer L_fg_ (30 μm) as an example, when T_h-BN_ is relatively thin, the floating gate is in closer proximity to the S/D electrodes, resulting in a stronger electrostatic coupling effect. This enhanced coupling weakens the vertical electric field between the floating gate and the MoS_2_ channel, thereby reducing the effectiveness of electron tunneling and leading to a lower MW. As T_h-BN_ increases, the electrostatic coupling effect is progressively mitigated, which in turn strengthens the vertical electric field, allowing for improved charge storage and an increase in MW. However, as T_h-BN_ continues to increase, the widening of the tunneling barrier eventually suppresses electron tunneling, leading to a gradual decrease in MW. This trend indicates that while an optimal T_h-BN_ can enhance memory performance by balancing the tunneling efficiency and electrostatic effects, excessive thickness ultimately hinders charge trapping due to increased energy barriers. These findings provide further evidence of the crucial role that electrostatic coupling plays in shaping charge storage behavior in FGM devices. Understanding the interplay between L_fg_, T_h-BN_, and electrostatic effects offers valuable insights for optimizing the tunneling layer design to achieve improved memory performance in low-dimensional memory devices.

**Figure 4:**
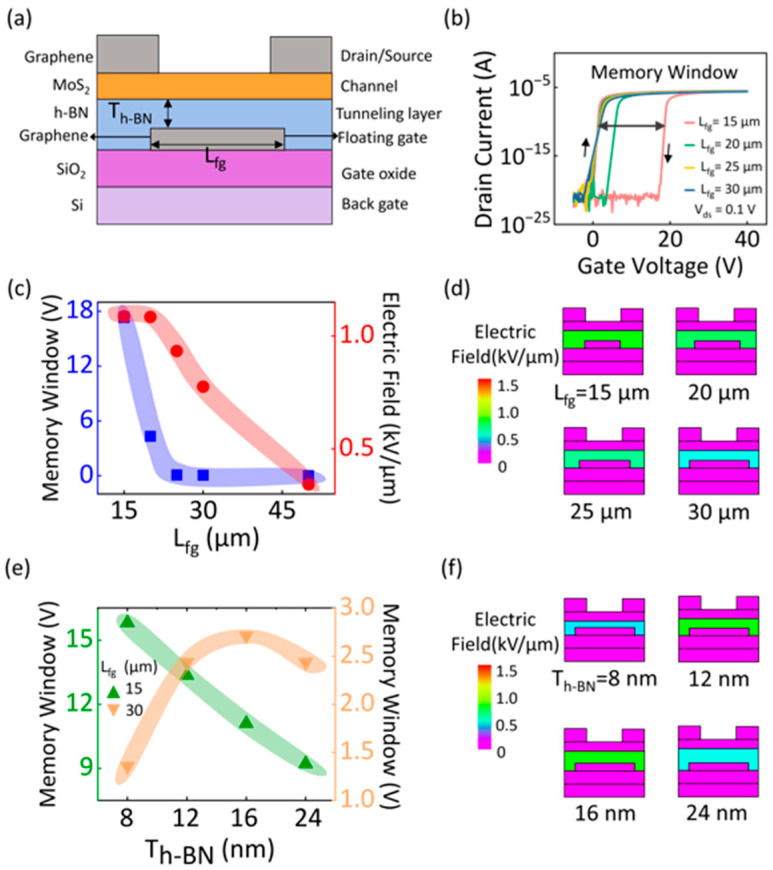
(**a**) The device structure and (**b**) recorded transfer curves for device simulation. (**c**) The evolution of the MW and electric field over the tunneling layer after programming as a function of L_fg_. (**d**) Simulated electric field distribution for devices with different L_fg_ (15, 20, 25, 30, and 50 μm). (**e**) The evolution of the MW over the tunneling layer after programming as a function of T_h-BN_ for the L_fg_ of 15 μm and 30 μm. (**f**) Simulated electric field distribution for devices with different T_h-BN_ (8, 12, 16, and 24 nm).

To further explore the underlying physical mechanisms of the critical role of floating-gate topology in shaping FGM charge storage capabilities, device modeling and electrical simulations were conducted using the ATLAS module of Silvaco TCAD. Note that due to the lack of several new materials in the database of the current simulation module, the used MoS_2_, h-BN, and Gr were newly defined according to the key physical parameters (Table 1) [41,42,43,44,45]. Figure 4a presents the simulated device structure, which shares the same architecture as the experimentally fabricated devices, ensuring consistency between the simulation and experimental conditions. The corresponding L_fg_-dependent MW characteristics are also depicted in Figure 4b,c, which revealed a clear monotonic decrease as L_fg_ increased from 15 μm to 50 μm. These results aligned well with the experimental observations and confirmed that increasing the L_fg_ distinctly suppresses the charge storage windows. To further reveal the dynamic tunability of L_fg_ on the charge storage behavior, the evolution of the electric field across the tunneling layer was simulated after the programming operation. As can be seen in Figure 4d, after programming, the electric field strength between the floating gate and the MoS_2_ channel showed a clear monotonic decline as L_fg_ increased. This phenomenon can be attributed to the intensification of electrostatic coupling effects, which reduces the vertical tunneling electric field and hinders efficient charge trapping. Note that this scenario is consistent with the physical images for the experimental results shown in Figure 2d, further validating the hypothesis that electrostatic coupling plays a critical role in memory performance. To gain insights into how the T_h-BN_ affects MW, further simulations were performed using two sets of devices aligning with our experiments, L_fg_ = 15 μm and L_fg_ = 30 μm, respectively. For devices with L_fg_ = 15 μm, the electrostatic coupling effects between the floating gate and S/D electrodes were relatively weak, and T_h-BN_ dominated during the carrier transport. As shown in Figure 4e, the simulated MW decreased monotonically as T_h-BN_ increased, which suggests that the thicker the tunneling barrier width (increasing T_h-BN_), the lower tunneling probability for carriers. However, for devices with L_fg_ = 30 μm, a distinct evolution of MW depending on T_h-BN_ could be observed. In this scenario, the MW initially increased as T_h-BN_ increased before reaching a peak and subsequently decreasing. The underlying mechanism can be explained by the competing influences of electrostatic coupling and tunneling barrier width. As the T_h-BN_ increases, the electrostatic coupling between the floating gate and S/D electrodes gradually weakens. This, in turn, leads to an enhancement in the vertical tunneling electric field between the floating gate and the channel, hence enhancing the charge storage capability and broadening the MW (Figure 4f). However, as the T_h-BN_ continues to increase, the widening of the tunneling barrier becomes dominant, affecting the charge storage. This would gradually weaken the tunneling electric field and in turn, negatively influence the electron tunneling probability and lead to a gradual decline in MW. These simulation results are consistent with the experimental findings, further confirming that floating-gate topology plays a crucial role in shaping the charge storage characteristics of FGM devices.

**Figure 5:**
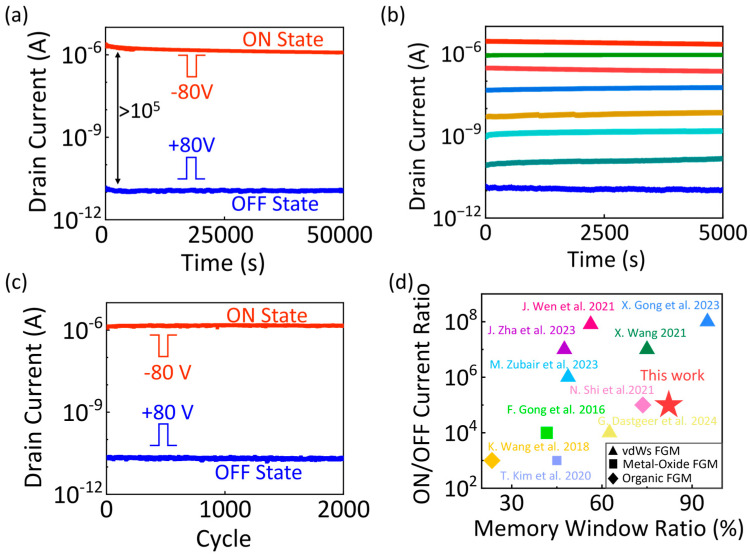
(**a**) Retention characteristic of the vdWs FGM under ON and OFF states with an operating gate voltage of −80 and 80 V, respectively (V_ds_ = 0.1 V). (**b**) Multi-bit storage function with 8 distinct states under different gate voltage pulses (from top to bottom: −80, 20, 25, 27, 29, 30, 31, and 80 V, pulse width = 1 ms). (**c**) Endurance characteristic of the vdWs FGM under cyclic programming and erasing operations (V_bg_ = ±80 V, 1 ms; V_ds_ = 0.1 V. (**d**) ON/OFF current ratio and MW ratio (the ratio of memory window size to swept voltage range) benchmark of the FGM devices [16,28,46,47,48,49,50,51,52].

Based on the principles of floating-gate configuration for FGM devices, we further performed fundamental device characterization to evaluate the memory characteristics of fabricated devices with an equal length of channel and floating gate. As shown in Figure 5a, the device demonstrated excellent retention, maintaining a high ON/OFF current ratio of over 10^5^ for 5×10^5^ s. For the 10 year linear extrapolation of the ON and OFF state currents, the ON state/OFF state ratio still exceeded 104 when the retention curves were extrapolated to 10 years, demonstrating the ultralong retention time of our memory device (Figure S3). Cycling endurance tests (Figure 5c) showed reproducible switching between low- and high-resistance states for 2000 cycles. Additionally, multi-bit storage functionality was achieved by regulating the programming process, producing a 3-bit storage characteristic with stable retention exceeding 5000 s (Figure 5b). Figure 5d further benchmarks the ON/OFF current ratio and MW ratio to make a comparison with previously reported FGM devices [16,28,46,47,48,49,50,51,52]. The results showed that our device achieved an ON/OFF ratio of 10^5^ and a MW ratio of 82.25%, which are comparable to those of FGM devices based on metal oxide semiconductors, organic materials, and other vdWs materials. These results underscore the reasonable device design principle of the floating-gate configuration in improving the memory performance of vdWs FGM devices.

## 4. Conclusions

In conclusion, we systematically investigated the mechanism underlying the impact of floating-gate topology on the MW of classical MoS_2_ FGM devices. Both the experimental measurements and simulation results revealed that the floating-gate length and the electrostatic coupling between the floating gate and S/D electrodes are critical factors influencing the charge storage capability. These findings highlight the significant role of floating-gate design in optimizing memory performance. The optimized device achieved remarkable electrical storage characteristics: a large memory window ratio (82.25%), good retention (>50,000 s, 8 states), and considerable endurance characteristics (>2000 cycles). This study provides fundamental insights into the role of floating-gate topology in vdWs FGM devices, offering valuable guidance for the design and fabrication of next-generation non-volatile memory technologies. These foundational studies on floating-gate topology provide critical guidance for the design and fabrication of next-generation high-performance vdWs FGM devices.

## Figures and Tables

**Table 1:** Supplementary parameters for various materials in Silvaco.

Material	Dopant Thickness	Value
MoS_2_	E_g_ (eV)	1.9
	ε_r_	4.2
	χ (eV)	4.7
	μ_n_ (cm^2^/(V·s))	200
	μ_p_ (cm^2^/(V·s))	76
Gr	E_g_ (eV)	0
	εr	25
	χ (eV)	4
	g_c_ (E)	3 × 10^17^
	g_v_ (E)	3 × 10^17^
	μ_n_ (cm^2^/(V·s))	1 × 10^4^
	μ_p_ (cm^2^/(V·s))	1 × 10^4^
h-BN	E_g_ (eV)	4
	ε_r_	7.5

## Data Availability

Data are contained within the article or Supplementary Materials.

## Supplementary Materials

The following supporting information can be downloaded at: https://www.mdpi.com/article/10.3390/nano15090666/s1, Figure S1: The output characteristic curves of the FGM; Figure S2: The Raman spectroscopy analysis of the MoS_2_/h-BN/Gr heterostructure; Figure S3: 10 years linear extrapolation of the ON and OFF state currents.
