# Supplementary material for: Investigating Floating-Gate Topology Influence on van der Waals Memory Performance
Source: Nanomaterials (Basel). 2025 Apr 27;15(9):666. doi: 10.3390/nano15090666 (PMC12073696; doi:10.3390/nano15090666)
Supplement: Supplementary file 1 [file nanomaterials-15-00666-s001.zip › nanomaterials-3539602-supplementary.pdf]

Supplementary Information

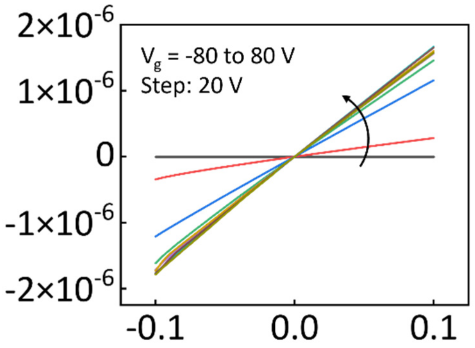

Supplementary Figure S1: The output characteristic curves of the FGM demonstrate good linearity, indicating excellent Ohmic contact between the Gr electrode and MoS_2.

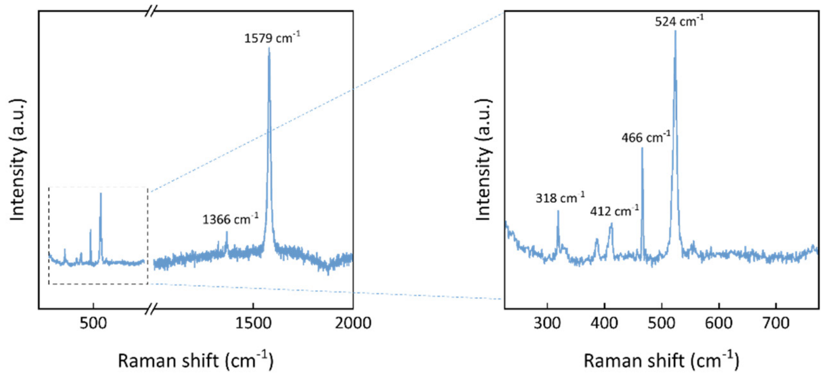

Supplementary Figure S2: The Raman spectroscopy analysis of the MoS_2/h-BN/Gr heterostructure showed that the E_{2g} peak of h-BN is located at 1366 cm^{-1}, the G peak of Gr at ~1579 cm^{-1}, and the four characteristic peaks of MoS_2 are distributed in the 318-412 cm^{-1} range, indicating good crystallinity of each layer in the material.

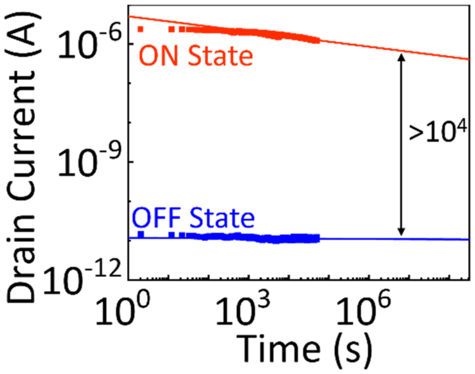

Supplementary Figure S3: 10 years linear extrapolation of the ON and OFF state currents.

The ON state/OFF state ratio still exceeds 10^4 when the retention curves are extrapolated to 10-year, demonstrating an ultralong retention time of our memory device.
